# *In Vitro* Antiplasmodial Activities and Synergistic Combinations of Differential Solvent Extracts of the Polyherbal Product, *Nefang*

**DOI:** 10.1155/2014/835013

**Published:** 2014-04-27

**Authors:** Protus Arrey Tarkang, Kathrin Diehl Franzoi, Sukjun Lee, Eunyoung Lee, Diego Vivarelli, Lucio Freitas-Junior, Michel Liuzzi, Tsabang Nolé, Lawrence S. Ayong, Gabriel A. Agbor, Faith A. Okalebo, Anastasia N. Guantai

**Affiliations:** ^1^Centre for Research on Medicinal Plants and Traditional Medicine, Institute of Medical Research and Medicinal Plants Studies (IMPM), P.O. Box 6163, Yaoundé, Cameroon; ^2^Department of Pharmacology and Pharmacognosy, University of Nairobi, P.O. Box 19676-00202, Nairobi 0800, Kenya; ^3^Early Drug Discovery Program, Institut Pasteur Korea, Republic of Korea; ^4^Centre for Neglected Diseases Drug Discovery, Institut Pasteur Korea, Republic of Korea

## Abstract

*Nefang*, a polyherbal product composed of *Mangifera indica* (bark and leaf), *Psidium guajava*, *Carica papaya*, *Cymbopogon citratus*, *Citrus sinensis*, and *Ocimum gratissimum* (leaves), is a potential therapy against *P. falciparum* malaria. *In vitro* antiplasmodial activities of its constituent solvent extracts were analyzed on CQ-sensitive (3D7) and multidrug resistant (Dd2) *P. falciparum* strains. The interactions involving the differential solvent extracts were further analyzed using a variable potency ratio drug combination approach. Effective concentration 50 (EC_50_) values were determined by nonlinear regression curve-fitting of the dose-response data and used in calculating the fractional inhibitory concentration 50 (FIC_50_) and combination indices (CI) for each pair. The derived EC_50_ values (3D7/Dd2, **μ**g/mL) are *Nefang*-96.96/55.08, *MiB*-65.33/34.58, *MiL*-82.56/40.04, *Pg*-47.02/25.79, *Cp*-1188/317.5, *Cc*-723.3/141, *Cs*-184.4/105.1, and *Og*-778.5/118.9. Synergism was obtained with *MiB/Pg* (CI = 0.351), *MiL/Pg* (0.358), *MiB/Cs* (0.366), *MiL/Cs* (0.482), *Pg/Cs* (0.483), and *Cs/Og* (0.414) when analyzed at equipotency ratios. Cytotoxicity testing of *Nefang* and the solvent extracts on two human cell lines (Hep G2 and U2OS) revealed no significant toxicity relative to their antiplasmodial activities (SI > 20). Taken together, our data confirm the antimalarial activities of *Nefang* and its constituent plant extracts and identified extract pairs with promising synergistic interactions for exploitation towards a rational phytotherapeutic and evidence-based antimalarial drug discovery.

## 1. Introduction

Since time immemorial, man searched for cures for his disease from nature. This ancient tradition of sourcing treatment from medicinal plants was initially instinctive [1] but has eventually resulted into traditional medicine being the first point of healthcare for many people around the world, especially where there is the absence of modern healthcare facilities [2]. Consequently, there has been an increased focus on medicinal plant research and a large amount of evidence has been collected to show its immense potential in various traditional health systems. In the last few years, studies have been carried out on a large number of plants, used by traditional healers for centuries [3], and some 7000 natural compounds isolated from these are currently used in modern medicine, thereby increasing the global market value of medicinal plant products. In spite of the development of pharmacological agents for the treatment of diseases, the use of medicinal plants continues to flourish. This continuous interest and perpetual use of medicinal plants has brought about today's modern and sophisticated procedures of their processing and use [4]. More than 90% of current therapeutic classes have been derived from a natural product prototype, whose discovery has led to significant changes in the practice of modern medicine [5, 6]. Phytotherapeutic products from these medicinal plants have become universally popular in primary healthcare, and some have been regarded as readily safe simply because they are of natural source. This presumption has led to plant products being widely used as self-medication without compromising health effects [7]. Nonetheless, the decreasing efficacy of synthetic drugs and the increasing contraindications of their use make the usage of herbal drugs topical again [8].

The dominant paradigm in drug discovery is the concept of designing maximally selective ligands to act on individual drug targets [9, 10]. This concept is not man-made but indirectly copied from nature. Selective pharmacological principles that occur obviously by chance in certain plants and animals have raised the awareness of the existence of such ligands.

Herbal drugs, singularly and/or in combinations, contain a myriad of compounds in complex matrices in which no single active constituent is responsible for the overall efficacy [11]. This constitutes some of the shortcomings in establishing quality control standards for raw materials and standardization of these herbal products. In spite of these, a large number of plant species have been identified through ethnobotanical and ethnopharmacological studies as potential sources of therapeutic agents and pure products (phytochemicals) with good activity have been isolated from some of them. Whole plants or parts of them are prepared and administered as oral decoctions, steam baths, infusion, or enemas. Most remedies are a concoction of two or more plant species that work in synergy [12], an example being* Nefang*, a polyherbal product that has been used for ages by the Bayang community of the southwest region of Cameroon for the treatment of malaria [13]. An earlier study evaluated the* in vitro* antioxidant and* in vivo *antioxidant properties of this polyherbal product [14] which may play a role in curbing oxidative stress related to malaria infection.

Malaria remains a major killer disease in sub-Sahara Africa, and the emerging resistance of the causative agents,* Plasmodium* spp., to most previously effective antimalarial drugs is a public health concern [15]. There is a growing consensus that drug combinations are essential to the optimal control of malaria, since they offer improved efficacy through synergistic activities [16]. Consequently, drug combination therapy, including the use of polyherbal products, has become the standard of care for* P. falciparum* drug resistance [17].

Pure drugs isolated from plants for their high activity have at times exhibited lesser activity than the crude extract at comparable dose or concentration of the active component [18]. This has been attributed to the presence of interacting substances present in the crude extract, resulting in higher activity than that of the isolated and purified component, a phenomenon which has been exploited in certain circumstances for the development of phytotherapeutic and conventional drugs. Therefore, there is a strong need for a valid complementary approach in herbal medicine research ethics [19], towards the standardization of crude polyherbal antimalarials with demonstrated safety and efficacy [20, 21].

This study aims at evaluating the* in vitro* antiplasmodial activities and characterizing the interactions between the constituent plant extracts of* Nefang,* a polyherbal product composed of* Mangifera indica* (bark and leaf) and the leaves of* Psidium guajava*,* Carica papaya*,* Cymbopogon citratus*,* Citrus sinensis, *and* Ocimum gratissimum*.

## 2. Materials and Methods

### 2.1. Collection and Extraction of Plant Material

Fresh parts of the constituent plants of* Nefang*, bark and leaves of* Mangifera indica* (*MiB* and* MiL*, resp.) and leaves of* Psidium guajava* (*Pg*),* Carica papaya* (*Cp*),* Cymbopogon citratus* (*Cc*),* Citrus sinensis *(*Cs*), and* Ocimum gratissimum* (*Og*), were harvested from their natural habitat in Cameroon between July and August 2011. Plant identification and voucher specimen referencing were done at the Institute of Medical Research and Medicinal Plants Studies (IMPM) Herbarium, Yaoundé, Cameroon, by a botanist. The freshly harvested plant parts were air-dried and pulverized, and aqueous and ethanol extraction of each plant material were carried out by percolation: weighed quantities (1000 g) of each plant part were exhaustively macerated in water (2.4 L) and ethanol (2.0 L), respectively, for 4 h. Each of the macerates was transferred into a conical percolator for 72 h and the extracts were filtered with a sieve of 80 *μ*m pore size [22]. Each ethanol filtrate was first concentrated using a rotary evaporator. Both filtrates were then concentrated in an air oven at 60°C. The extracts were weighed and stored in labeled sealed plastic containers at 4°C until being used to prevent contamination.

### 2.2. Preliminary Phytochemical Screening

The constituent plant extracts were analyzed for the presence of alkaloids, anthocyanins, flavonoids, phenols, saponins, tannins, triterpenes, and sterols according to standard methods [23, 24].

### 2.3. Cytotoxicity Screening

Cytotoxicity screening of* Nefang* and its constituent plant extracts was carried out using the Resazurin Fluorometric Cell Viability Assay method [25, 26] on Hep G2 hepatoma and U2OS osteosarcoma epithelial cell lines (provided by Institut Pasteur, Korea). All the chemicals were ordered from Sigma-Aldrich Inc. (Germany). The cells were maintained in Dulbecco's MEM glutamax-1 containing sodium pyruvate, glucose, and pyridoxine and supplemented with 10% FCS. 200 mg/mL of each ethanol extract was prepared in 100% DMSO and diluted 1/10 in DMEM to obtain 20 mg/mL in 10% DMSO. Meanwhile 20 mg/mL of each aqueous extract was prepared in DMEM. Serial dilutions were prepared for each extract in an intermediate DRC plate (384-well format) containing 25 *μ*L of 10% DMSO (ethanol extracts) or 25 *μ*L of DMEM (aqueous extracts) to obtain concentrations of 2000 *μ*g/mL–0.061 *μ*g/mL. Each dose-response experiment comprised a 2-fold dilution of the extracts (2000 *μ*g/mL max concentration, 16 dose-response points) in DMSO (Cf < 1% ethanol extracts) or plain DMEM. Hep G2 hepatoma and U2OS osteosarcoma epithelial cells in log phase of growth were harvested by trypsinization (0.05% trypsin-treatment for 10 min) and then seeded at 5 × 10^3^ cells/per 100 *μ*L of media in a DRC 384-well plate followed by a 24-hour culture at 37°C in a 5% CO_2_ incubator to allow for cell attachment. To each well, 10 *μ*L of each concentration of plant extract was added in triplicate. Each plate contained an untreated cell control, a blank control, and puromycin standard. Prepared plates were incubated at 37°C for 72 h in a 5% CO_2_ environment. After incubation, 10 *μ*L of resazurin solution was added to each well and plates were incubated for further 12 h. Fluorescence of the formed resorufin product in each well was measured with excitation wavelength at 530 nm and emission wavelength at 590 nm using a VICTOR microtiter plate reader. Fluorescence signal from each sample was obtained after background fluorescence subtraction.

### 2.4. Evaluation of* In Vitro* Antiplasmodial Activity

*In vitro* susceptibility assays of* Nefang* and its constituent plant extracts were performed on cultured 3D7 (MRA-102, CQ-sensitive) and Dd2 (MRA-156, MDR) strains of* Plasmodium falciparum* [27]. The parasite strains 3D7 and Dd2 were kindly donated by the Biodefense and Emerging Infections (BEI) Research Resources (MR4, Manassas, VA, USA) and maintained in continuous culture, with back-up stored in liquid nitrogen. All the chemicals except Albumax II (Gibco, Invitrogen, USA) were ordered from Sigma-Aldrich Inc. (Germany). The laboratory strains of* P. falciparum *were grown and maintained in culture under microaerophilic conditions using the method described by Trager and Jensen [28] with the following modifications: both parasite strains were maintained at 3% hematocrit in human red blood cells (blood type A+, Gyeonggi Blood Center, Korean Red Cross) in media comprising RPMI 1640, 25 mM HEPES buffer (pH 7.4), 100 *μ*M hypoxanthine, 16 *μ*M thymidine, 20 *μ*g/mL gentamycin, and 0.5% Albumax. Cultures were grown at 37°C in 75-cm^2^ flasks after gassing with a mixture of 5% CO_2_, 1% O_2_, and 94% N_2_. Parasites were double-synchronized (8-hour interval) by 5% sorbitol-treatment at the ring-stage and then cultivated for one complete developmental cycle prior to the assays [29].

*In vitro* extract activity on 3D7 and Dd2 strains of* P. falciparum *was determined by the SYBR-Green 1 fluorescence-based method [30]. 200 mg/mL of each ethanol and 20 mg/mL of each aqueous extract were prepared as earlier described, replacing DMEM with RPMI 1640. The ring-stage parasitized erythrocytes (~10 hpi) were diluted in fresh blood and complete culture medium to 0.5% parasitaemia and 1.5% hematocrit, respectively, and 45 *μ*L was added using a WellMate liquid handler, to 5 *μ*L extract preparation in a 384-well microtiter plate (Greiner, black) and in triplicate. Control wells comprised infected RBC in culture media alone (positive growth controls), uninfected RBC at 1.5% hematocrit (background controls), and the antimalarial drugs chloroquine and artemisinin as treatment controls. The plates were then assembled in culture chambers, gassed, and incubated at 37°C for 72 h prior to SYBR-Green I fluorescence-based assay. Parasite growth was monitored microscopically with a Giemsa stained thin blood smear from a tracking culture and the experiment was terminated when the untreated parasites had reached the early trophozoite stage of the second cycle.

Three times SYBR-Green 1 assay: lysis solution (for 12 mL) was prepared by adding 300 *μ*L of Tris base (1 M), 180 *μ*L of EDTA (500 mM), 9.6 *μ*L of saponin (15%), and 14.4 *μ*L of Triton X-100 (100%) to 11.436 mL of Milli-Q water. Just before use 0.3 *μ*L of SYBR-Green I (10,000x) was added per mL of lysis solution.

By using a WellMate liquid handler, 25 *μ*L of lysis/SYBR-Green I solution was added directly to each 50 *μ*L culture in the 384-well microtiter plates and sealed with Platemax sealing film. Each plate was vortexed using a MixMate vortexer for 45 sec at 1700 rpm and then wrapped with aluminum foil and incubated at room temperature for 1 h prior to fluorescence reading using a VICTOR microtiter plate reader (Ex/Em: 485 nm/530 nm).

### 2.5. Characterization of the Interaction between Solvent Extracts of* Nefang*

*In vitro* susceptibility assays of paired constituent plant extracts of* Nefang* were performed on cultured Dd2 (MDR) strain of* Plasmodium falciparum* using the fixed-ratio drug combination method [31] as follows.

Paired combinations of aqueous and ethanol extracts of the constituent plants of* Nefang* were prepared at equipotency ratios (5EC_50_A : 5EC_50_B) from a stock of 20 mg/mL. Two-fold serial dilutions were then prepared in an intermediate 384-well DRC plate containing 5% DMSO (ethanol-containing extract pairs) or plain RPMI 1640 (aqueous extract pairs) to obtain 16 dose-response points. The ring-stage parasitized erythrocytes (~10 hpi) were then diluted in complete medium to 0.5% parasitaemia and 1.5% hematocrit and 45 *μ*L was added in triplicate to 5 *μ*L extract pair preparation in a 384-well plate (Greiner, black), using a WellMate liquid handler. Control wells comprised infected RBC (positive growth controls), uninfected RBC at 1.5% hematocrit (background controls), and the antimalarial drug combinations chloroquine/chloroquine and chloroquine/artemisinin at equipotency ratios as drug-drug interaction controls. The plates were then assembled in culture chambers, gassed, and incubated at 37°C for 72 h prior to SYBR-Green I fluorescence-based assay. Parasite growth was monitored microscopically with a Giemsa stained thin blood smear from a tracking culture and the experiment was terminated when the untreated parasites had reached the early trophozoite stage of the second cycle.

Following the above assays, extract combinations that demonstrated promising synergistic or additive interactions were selected and further analyzed using a variable potency ratio drug combination approach starting at 5EC_50_A : 0EC_50_B to 0EC_50_A : 5EC_50_B paired combinations. Parasite growth in the plate wells was then assessed using the SYBR-Green I fluorescence-based assay as described earlier.

### 2.6. Selectivity Index

Selectivity indices (SI = CC_50_/EC_50_) [32] were calculated for each extract as an indication of its toxicity relative to the observed antimalarial activity.

Furthermore, the obtained EC_50_s were used to calculate 50% fractional inhibitory concentrations (FIC_50_) and combination indices as previously described [33, 34].

That is, FIC_50_A = EC_50_ of drug A in combination/EC_50_ of drug A alone.

The sums of the FIC_50_ gave the combination index (CI) of the pair (CI_A/B_ = FIC_50_A + FIC_50_B). For CI values, sums of less than 1.0 (CI < 1) represented a trend toward synergy and greater than 1.0 (CI > 1) represented a trend toward antagonism.

FIC_50_s of drug A and drug B at different combination ratios were used to plot isoboles, with the line of additivity running from point (0,1) of the vertical axis to point (1,0) of the horizontal axis. Synergy or antagonism was revealed when the plotted FIC_50_ values were below or above the line of additivity, respectively.

### 2.7. Statistical Analysis

To determine 50% cytotoxic (CC_50_) and effective (EC_50_) concentration values for each extract or extract combinations, the obtained data were analyzed using GraphPad Prism 6.0. The logarithm of the extract concentration was plotted against its activity represented by the fluorescence reading to obtain a nonlinear regression curve-fitting and a variable slope sigmoidal dose-response curve.

## 3. Results

### 3.1. Extraction of Plant Material

The common names, place of collection, voucher specimen number, and yields of the aqueous and ethanol extracts of the constituent plants of* Nefang* are shown in Table 1. The ethanol extract of* Mangifera indica* leaves had the highest yield while the aqueous extract of* Citrus sinensis* had the lowest. However, for each plant part, the ethanol extract had a higher yield than the corresponding aqueous extract.

### 3.2. Preliminary Phytochemical Screening

Preliminary phytochemical screening of the constituent plant extracts of* Nefang* revealed the presence of flavonoids, phenols, triterpenes, and sterols in all extracts, saponins in all except* Cc*, tannins in all except* Cp* and* Cc,* and alkaloids in* MiB* and* Og* only (Table 2).

### 3.3. Cytotoxicity Screening and Evaluation of* In Vitro* Antiplasmodial Activity

All the aqueous and ethanol extracts were screened against Hep G2 hepatoma and U2OS osteosarcoma epithelial cell lines and the results showed no significant or toxic activity (SI > 20) of* Nefang* and the majority of its component extracts (Table 3).

The* in vitro* antiplasmodial activity of all the aqueous and ethanol extracts of the constituent plants of* Nefang* against cultured 3D7 (CQ-sensitive) or Dd2 (MDR) strains of* Plasmodium falciparum* is summarized in Table 3. Of the 16 extracts tested, 9 showed significant antiplasmodial activity at concentrations less than 50 *μ*g/mL. EC_50_s (3D7/Dd2) of the ethanol extracts exhibiting good antiplasmodial activities against both parasite strains were* MIB*-24.46/14,* MIL*-24.32/16.34,* Pg-*37.28/23,* Cc-*28.75/54.84, and* Nefang*-51.10/29.99 (*μ*g/mL) whereas the only aqueous extract with a similarly promising antiplasmodial activity was* Pg*-47.02/25.79 *μ*g/mL. All other extracts revealed weak activities (EC_50_ > 100 *μ*g/mL) against one or both parasite strains, indicating that not all solvent extracts of* Nefang* exhibited antimalarial properties.

### 3.4. Characterization of the Interaction between Solvent Extracts of* Nefang*

For stringency reasons, the interactions between the various plant extracts at equipotency ratios as evaluated in this study were classified as synergistic (CI < 0.7), additive (0.7 < CI < 1.5), or antagonistic (CI > 1.5). Additionally, fold increases in the extracts' activities in a pair, relative to the activities when tested alone, were determined and used to identify pairs not exhibiting synergistic, additive, or antagonistic interactions. Thus,* Cp/Og*-(EtOH)—(CI = 0.36),* MiB/Pg*-(Aq)—(CI = 0.35),* MiB/Cs*-(Aq)—(0.36),* MiL/Pg*-(Aq)—(0.36),* Cp/Cs*-(Aq)—(0.30), and* Cc/Cs*-(Aq)—(0.32) were identified as exhibiting strong apparent synergism with antiplasmodial activities >5-fold that of* Nefang* or the respective activities when tested alone (Table 4). The combinations exhibiting apparent additive or antagonistic interactions are indicated in Tables 5 and 6, respectively.

**Figure 1:**
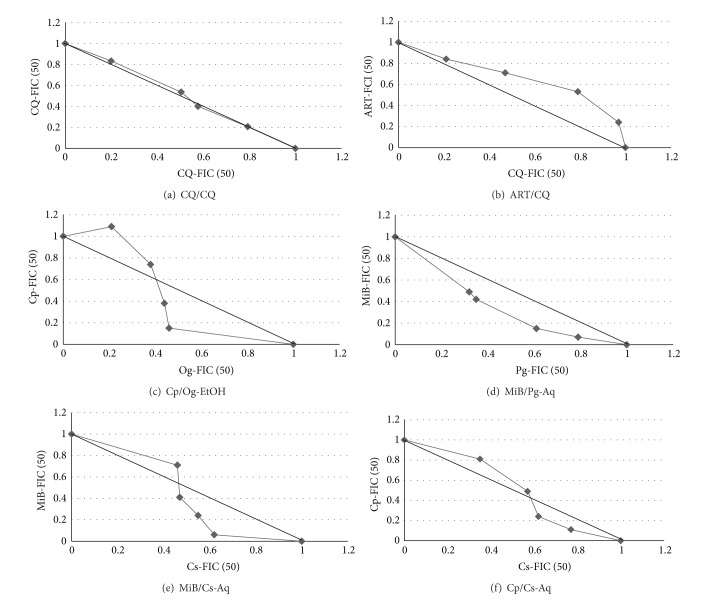
Isobolograms of the* in vitro* interactions between differential solvent extracts of* Nefang *at variable potency ratios. (a): Control CQ/CQ; (b): Control ART/CQ; (c): Cp/Og-EtOH; (d): MiB/Pg-Aq; (e): MiB/Cs- Aq; (f): Cp/Cs-Aq.* CQ: Chloroquine; ART: Artemisinin; *FIC_50_ = Fractional Inhibitory Concentration 50. A concave isobologram is consistent with synergy, a convex one is consistent with antagonism, and a straight line is consistent with additivity. Axes are EC_50_s normalized to 1.

As shown in Figure 1, isobole analyses of extract pairs with apparent synergistic interactions confirmed the occurrence of synergism over a wide range of combination ratios in 4 of the 6 identified pairs. These include* Cp/Og*-EtOH (ratios of 2 : 3 to 1 : 4),* MiB/Pg*-Aq (4 : 1 to 1 : 4),* MiB/Cs*-Aq (3 : 2 to 1 : 4), and* Cp/Cs*-Aq (2 : 3 to 1 : 4).

## 4. Discussion

The CC_50_ of all the aqueous and ethanol extracts on both Hep G2 and U2OS cell lines was above 2000 *μ*g/mL indicating safety of* Nefang*. This safety has been earlier confirmed in previous* in vivo* toxicity studies reported on* M. indica *bark [35] and leaves of* M. indica* [36],* P. guajava* [37],* C. papaya* [38],* C. citratus* [39],* C. sinensis* [40], and* O. gratissimum* [41] as well as* Nefang* (unpublished).

Based on WHO and other previous reports,* in vitro* antiplasmodial activity is considered as good when EC_50_ < 50 *μ*g/mL [42, 43]. The best antiplasmodial activities (twofold greater activity than* Nefang*) were obtained with ethanol extracts of* M. indica *bark and leaves, whereas* C. papaya* and* O. gratissimum *were the least active. These findings are consistent with previous observations by Bidla et al. [44] for* M. indica* and* C. citratus*, Ngemenya et al. [45] for* C. papaya* and* O. gratissimum,* and Nundkumar and Ojewole [46] for* P. guajava*. This once more confirms the antimalarial activities of some herbal extracts used in traditional medicine. Inasmuch as plant extracts singly or in combination have been increasingly evaluated for their* in vitro* antiplasmodial activities, our study is the first to demonstrate the* in vitro* antiplasmodial activity of* Nefang* and its component plants (singly and in paired combinations) using both CQ-sensitive and MDR* Plasmodium* parasites.

Various parameters such as localization and period of collection, plant part, drying procedure, and extract preparation may modify the pharmacological response produced by a single species. Phytochemical screening of the constituent plants of* Nefang* revealed the presence of alkaloids, anthocyanins, flavonoids, phenols, saponins, tannins, triterpenes, and sterols. These results are consistent with previous results from a review of the biological activity and chemical analyses of extracts of the component plants of* Nefang *[13]. Alkaloids are one of the major antimalarial natural products and various classes have been reported to exhibit promising activities [47]. Quinine, an illustrative example, was one of the first widely used antimalarial drugs due to its parasite DNA intercalating property, possessed by many other classes. It has, however, fallen into disuse due to emerging parasite strains resistant to the drug. Consequently, it has been replaced by more effective synthetic drugs derived from the acridine and quinoline structure, such as chloroquine and mefloquine, which inhibit heme polymerase and prevent the polymerization of heme to hemozoin, thereby causing oxidative-metabolic effects on the parasite, and primaquine which destroys the gametocytes of malaria parasites [48]. Some nonalkaloidal natural products such as terpenes, flavonoids, and their related compounds have also been reported to exhibit promising antiplasmodial activities [49]. Triterpenoids such as iridal extracted from* Iris germanica* L. are suspected to act against the reinvasion step rather than the maturation step of* P. falciparum* and have cumulative inhibitory effect on the main metabolic pathways of the parasite [50]. Inasmuch as the mechanism of action of flavonoids is unclear, some flavonoids have been shown to inhibit the influx of L-glutamine and myoinositol into* P. falciparum*-infected erythrocytes [51], while others such as a flavone glycoside from* Phlomis brunneogaleata* and iridoid from* Scrophularia lepidota* have been reported to inhibit the FabI enzyme of* P. falciparum* [52, 53].

Investigations on the efficacy of antimalarial plants usually focus on killing the parasite but rarely consider other mechanisms. Many of these herbal remedies exert their anti-infective effects not only directly on the pathogen but also indirectly stimulating the natural and adaptive defense mechanisms of the host, thereby suppressing or eliminating the parasite [54]. Therefore, some of the nonantiplasmodial secondary metabolites such as phenols could mitigate malaria parasite infection in the host by conferring a protective/antioxidative effect against oxidative stress induced in the host parasitized red blood cells by the malaria parasite [48]. These results confirm that these active and nonantiplasmodial components are responsible for the overall antimalarial activity of* Nefang*.

The potent antiplasmodial activities and weak cytotoxicity profiles of most of the extracts in this study suggest high selectivity for* P. falciparum*. The reasonably high SI values for the extracts indicate that smaller quantities of the active component will be needed to achieve high clinical efficacy with increased tolerability and safety [31]. The effectiveness of any plant extract is dependent upon a favorable therapeutic ratio; that is, the drug must kill or inhibit the parasite but must have little or no toxicity to the host. The selectivity of a plant to inhibit the growth of a parasite and yet be less toxic to the host depends on differences in biochemistry between the parasite and the host. Such a plant could operate on a biochemical target in the parasite that is either absent or significantly different in the host [55].

Our interaction studies with various pairs of the differential solvent extracts of the constituent plants revealed the presence of twenty-three synergistic, seventeen additive, and five antagonistic pairs at equipotency. These results are consistent with previous observations by Azas et al. [56] and Gathirwa et al. [57], on different solvent plant extracts. Among the synergistic pairs, only six showed promising activity. These interactions were further demonstrated by isobologram analysis at variable potency ratios, wherein* MiB/Pg*-(Aq) exhibited outright synergistic interaction at all experimental concentrations, while* Cp/Og*-(EtOH),* MiB/Cs*-(Aq), and* Cp/Cs*-(Aq) exhibited antagonistic and synergistic interactions as their concentrations were inversely varied, respectively.* In vitro* sensitivity assessment of drug combinations for malaria is used to help predict clinically useful combinations. Theoretically,* in vitro* synergy signifies that less than 50% of each of the components should achieve 100% therapeutic rates. The greater the synergy, the less the amount required of each component. Therefore, reduced doses of one or both components may lead to increased tolerability and safety, more practical dosing regimens, and/or decreased cost. Additionally, synergy may allow two drugs, both less than 50% efficacious, to be combined to achieve a very high efficacy [31]. On the other hand,* in vitro* antagonism signifies that more than 50% of each of the components will be needed to achieve maximum therapy. The greater the antagonism, the larger the amount required of each component.

Some of the component extracts were inactive against both parasite strains and showed low SI values and high CI values in combination. This suggests the presence of weakly active and/or antagonizing components whose interactions with the active constituents could mitigate paired activity and/or overall antimalarial activity of* Nefang*. These antagonizing components could be nontoxic to the host and nontoxic to the parasite as well. Thus, eliminating such undesirable components in* Nefang* or selectively combining the active extracts might increase overall activity and tolerability as suggested elsewhere [58]. Therefore, understanding the modes of interaction between the individual plant components would be of immense importance for the identification of compounds and/or mixtures for downstream clinical development.

## 5. Conclusion

The* in vitro* antiplasmodial activity of* Nefang* has been demonstrated and we hope this therapeutic assessment of constituent extracts and their combinations would assist in developing combinations with optimum efficacy for further* in vivo* analyses and exploitation towards a rational antimalarial phytotherapeutic drug discovery. Additionally, it is expected that the antiplasmodial components of* Nefang* would interact positively with conventional antimalarial compounds, thereby potentiating their activity in resistant parasite strains.

## Figures and Tables

**Table 1:** Constituent plants of *Nefang*: voucher numbers, common names, parts used, collection, and extraction yield.

Plant family and species (voucher specimen number)	Common name (part used)	Place of harvest	Extraction	
			Ethanol yield (%)	Aqueous yield (%)
Anacardiaceae *Mangifera indica* Linnaeus (TN6225)	Mango (bark and leaves)	Mballa II, Yaoundé	5.40 and 8.05^∗#^	5.52 and 6.20

Myrtaceae *Psidium guajava* Linnaeus (TN6226)	Guava (leaves)	Nkomo, Yaoundé	7.88	5.84

Caricaceae *Carica papaya* L. papaya (TN6227)	Pawpaw (leaves)	Nkoabang, Yaoundé	7.94	6.59**

Poaceae *Cymbopogon citratus* (DC. Ex Nees) Stapf (TN6228)	Lemon grass or fever grass	Kombone, Kumba	6.70	5.80

Rutaceae *Citrus sinensis* (Linnaeus) Osbeck (pro sp.) [maxima reticula] (TN6229)	Sweet orange (leaves)	Mamfe	4.85	3.28

Lamiaceae *Ocimum gratissimum* Linnaeus (TN6230)	Wild basil or mosquito plant (leaves)	Buea	5.63	4.64

^#^Highest extraction yield; *highest ethanol extraction yield; **highest aqueous extraction yield.

**Table 2:** Phytochemical screening of the constituent plants extracts of *Nefang*.

Phytochemical constituent	Plant						
	*MiB *	*MiL *	*Pg *	*Cp *	*Cc *	*Cs *	*Og *
Alkaloids	+	−	−	−	−	−	+
Anthocyanins	+	+	+	−	−	−	+
Flavonoids	+	+	+	+	+	+	+
Phenols	+	+	+	+	+	+	+
Saponins	+	+	+	+	−	+	+
Tannins	+	+	+	−	−	+	+
Triterpenes and sterols	+	+	+	+	+	+	+

+: presence; −: absence.

**Table 3:** Cytotoxicity profile (CC_50_), *in vitro* antiplasmodial activity (EC_50_), selectivity index (SI) of *Nefang* and constituent plant extracts.

Nature of extract	Extract	CC_50_ (µg/mL) U2OS	CC_50_ (µg/mL) Hep G2	EC_50_ 3D7 (µg/mL)	EC_50_ Dd2 (µg/mL)	Selectivity index (SI = CC_50_-Hep G2/EC_50_-Dd2)
Ethanol (EtOH)	*Mangifera indica *bark	>2000	>2000	24.46 ± 0.03	14.00 ± 0.03**	>142.85*
	*Mangifera indica *leaf	>2000	>2000	24.32 ± 0.03	16.34 ± 0.04	>122.39*
	*Psidium guajava *	>2000	>2000	37.28 ± 0.02	23.00 ± 0.03	>86.95*
	*Carica papaya *	>2000	>2000	76.03 ± 0.04	121.60 ± 0.11	>16.44
	*Cymbopogon citratus *	>2000	>2000	28.75 ± 0.04	54.84 ± 0.01	>36.47
	*Citrus sinensis *	>2000	>2000	39.34 ± 0.04	86.08 ± 0.14	>23.34
	*Ocimum gratissimum *	>2000	>2000	81.46 ± 0.04	121.50 ± 0.08	>16.46
	*Nefang *	**>2000**	**>2000**	**51.10 **± 0.02	**29.99 **± 0.04	**>68.44**

Aqueous (Aq)	*Mangifera indica *bark	>2000	>2000	65.33 ± 0.02	34.58 ± 0.03	>57.84*
	*Mangifera indica *leaf	>2000	>2000	82.56 ± 0.02	40.04 ± 0.03	>49.95*
	*Psidium guajava *	>2000	>2000	47.02 ± 0.03	25.79 ± 0.03	>77.56*
	*Carica papaya *	>2000	>2000	1188.00 ± 0.03	317.50 ± 0.09	>6.29
	*Cymbopogon citratus *	>2000	>2000	723.30 ± 0.01	141.00 ± 0.07	>14.18
	*Citrus sinensis *	>2000	>2000	184.40 ± 0.04	105.10 ± 0.08	>19.03
	*Ocimum gratissimum *	1872.5	>2000	778.50 ± 0.10	118.90 ± 0.09	>16.82
	*Nefang *	**>2000**	**>2000**	**96.96 **± 0.03	**55.08 **± 0.03	**>36.31**

Standard drugs	Chloroquine	—	—	21.0 ± 0.01 nM	139.60 ± 0.05 nM	—
	Artemisinin	—	—	20.63 ± 0.01 nM	18.20 ± 0.04 nM	—

EC_50_ 3D7/Dd2 expressed as mean ± SEM, *n* = 3.

*SI (extract) > SI (*Nefang*): potentially safer and promising therapy; **best antiplasmodial activity.

**Table 4:** *In vitro* antiplasmodial activity (EC_50_) of paired extracts exhibiting synergistic interaction (CI < 0.7) at equipotency ratios.

Number	Extract combination	EC_50_ ratio (µg/mL)	EC_50_ ratio in combination (µg/mL)	FIC_50_ A	FIC_50_ B	Fold increase	Combination index (CI)
1	Cp/Cc-EtOH	121.60/54.84	28.45/12.83	0.24	0.24	4.27/4.27	0.48
2	Cp/Cs-EtOH	121.60/86.08	37.69/26.68	0.31	0.31	3.23/3.23	0.62
3	**Cp/Og-EtOH**	**121.60/121.5**	**21.39/21.46**	**0.18**	**0.18**	**5.68/5.66**	0.36*
4	Cc/Og-EtOH	54.85/121.5	13.68/30.43	0.25	0.25	4.01/3.99	0.50
5	Cs/Og-EtOH	86.08/121.5	21.99/30.17	0.26	0.26	3.91/4.02	0.52
6	MiL-EtOH /MiL-Aq	16.34/40.04	5.26/12.88	0.32	0.32	3.11/3.11	0.64
7	Pg-EtOH/Pg-Aq	23/25.79	5.99/6.72	0.26	0.26	3.84/3.84	0.52
**8**	**MiB/Pg-Aq**	**34.58/25.79**	**6.07/4.53**	**0.17**	**0.17**	**5.70/5.70**	0.35*
9	MiB/Cp-Aq	34.58/317.5	10.24/93.99	0.29	0.29	3.38/3.38	0.59
**10**	**MiB/Cs-Aq**	**34.58/105.1**	**6.34/19.25**	**0.18**	**0.18**	**5.45/5.46**	0.36*
**11**	**MiL/Pg-Aq**	**40.04/25.79**	**7.18/4.62**	**0.18**	**0.18**	**5.58/5.58**	0.36*
12	MiL/Cp-Aq	40.04/317.5	11.59/91.91	0.29	0.29	3.45/3.45	0.58
13	MiL/Cs-Aq	40.04/105.1	9.63/25.27	0.24	0.24	4.16/4.16	0.48
14	Pg/Cp-Aq	25.79/317.5	6.88/84.64	0.27	0.27	3.75/3.75	0.54
15	Pg/Cc-Aq	25.79/141	8.26/45.13	0.32	0.32	3.12/3.12	0.64
16	Pg/Cs-Aq	25.79/105.1	6.22/25.36	0.24	0.24	4.15/4.14	0.48
17	Pg/Og-Aq	25.79/118.90	7.52/34.66	0.39	0.39	3.43/3.43	0.58
18	Cp/Cc-Aq	317.50/141	80.11/35.57	0.25	0.25	3.96/3.96	0.50
**19**	**Cp/Cs-Aq**	**317.50/105.10**	**46.50/15.39**	**0.15**	**0.15**	**6.83/6.83**	0.30*
20	Cp/Og-Aq	317.50/118.90	107.4/40.21	0.34	0.34	2.96/2.96	0.68
**21**	**Cc/Cs-Aq**	**141/105.10**	**22.19/16.54**	**0.16**	**0.16**	**6.35/6.35**	0.32*
22	Cc/Og-Aq	141/118.90	48.93/41.26	0.35	0.35	2.88/2.88	0.70
23	Cs/Og-Aq	105.10/118.90	21.78/24.64	0.21	0.21	4.83/4.83	0.42

Results presented as mean,  *n* = 3; *extract pairs exhibiting strong synergistic interactions.

EtOH: ethanol; Aq: aqueous.

**Table 5:** *In vitro* antiplasmodial activity (EC_50_) of paired extracts exhibiting additive interaction (0.7 < CI < 0.7) at equipotency ratios.

Number	Extract combination	EC_50_ ratio (µg/mL)	EC_50_ ratio in combination (µg/mL)	FIC_50_ A	FIC_50_ B	Fold increase	Combination index (CI)
1	MiB/MiL-EtOH	14/16.34	6.88/8.02	0.49	0.49	2.03/2.03	0.98
2	MiB/Cp-EtOH	14/121.60	7.99/69.36	0.57	0.57	1.75/1.75	1.14
3	MiB/Og-EtOH	14/121.50	5.20/45.32	0.37	0.37	2.69/2.68	0.74
4	MiL/Pg-EtOH	16.34/23	7.94/11.18	0.48	0.48	2.06/2.06	0.96
5	MiL/Cc-EtOH	16.34/54.84	11.01/36.94	0.67	0.67	1.48/1.48	1.34
6	MiL/Cs-EtOH	16.34/86.08	9.22/48.55	0.56	0.56	1.77/1.77	1.12
7	MiL/Og-EtOH	16.34/121.50	5.89/43.95	0.36	0.36	2.77/2.76	0.72
8	Pg/Cp-EtOH	23/121.60	10.85/57.38	0.47	0.47	2.12/2.12	0.94
9	Pg/Cs-EtOH	23/86.08	13.77/51.52	0.59	0.59	1.67/1.67	1.19
10	Pg/Og-EtOH	23/121.50	11.31/59.97	0.49	0.49	2.03/2.03	0.98
11	Cc/Cs-EtOH	54.84/86.08	35.03/54.99	0.64	0.64	1.57/1.57	1.28
12	MiB-EtOH/MiB-Aq	14/34.58	7.75/19.13	0.55	0.55	1.81/1.81	1.10
13	MiB/MiL-Aq	34.58/40.04	19.68/22.78	0.57	0.57	1.76/1.76	1.14
14	MiB/Cc-Aq	34.58/141	16.87/68.79	0.49	0.49	2.05/2.05	0.98
15	MiB/Og-Aq	34.58/118.90	14.91/51.28	0.43	0.43	2.32/2.32	0.86
16	MiL/Cc-Aq	40.04/141	14.31/50.39	0.36	0.36	2.80/2.80	0.72
17	MiL/Og-Aq	40.04/118.90	16.95/50.32	0.42	0.42	2.36/2.36	0.84

Results presented as mean, *n* = 3.

EtOH: ethanol; Aq: aqueous.

**Table 6:** *In vitro* antiplasmodial activity (EC_50_) of paired extracts exhibiting antagonistic interaction (CI > 1.5) at equipotency ratios.

Number	Extract combination	EC_50_ ratio (µg/mL)	EC_50_ ratio in combination (µg/mL)	FIC_50_ A	FIC_50_ B	Fold increase	Combination index (CI)
1	MiB/Pg-EtOH	14/23	16.77/27.55	1.20	1.20	0.83/0.83	2.40
2	MiB/Cc-EtOH	14/54.84	24.85/97.33	1.78	1.78	0.56/0.56	3.56
3	MiB/Cs-EtOH	14/86.08	10.86/66.79	0.78	0.78	1.29/1.29	1.56
4	MiL/Cp-EtOH	16.34/121.60	12.33/91.78	0.75	0.75	1.33/1.33	1.50
5	Pg/Cc-EtOH	23/54.84	24.47/58.34	1.06	1.06	0.94/0.94	2.12

Results presented as mean, *n* = 3.

EtOH: ethanol; Aq: aqueous.
